# Deep learning-based prediction of future growth potential of technologies

**DOI:** 10.1371/journal.pone.0252753

**Published:** 2021-06-04

**Authors:** June Young Lee, Sejung Ahn, Dohyun Kim

**Affiliations:** 1 Future Technology Analysis Center, Korea Institute of Science and Technology Information, Seoul, Korea; 2 Department of Industrial and Management Engineering, Myongji University, Yongin, Korea; Vellore Institute of Technology: VIT University, INDIA

## Abstract

Research papers are a repository of information on the various elements that make up science and technology R&D activities. Generating knowledge maps based on research papers enables identification of specific areas of scientific and technical research as well as understanding of the flow of knowledge between those areas. Recently, as the number of electronic publishing and informatics archives along with the amount of accumulated knowledge related to science and technology has proliferated, the need to utilize the meta-knowledge obtainable from research papers has increased. Therefore, this study devised a model based on meta-knowledge (i.e., text information including citations, abstracts, area codes) for prediction of future growth potential using deep learning algorithms and investigated the applicability of the various forms of meta-knowledge to the prediction of future growth potential. It also proposes how to select the promising technology clusters based on the proposed model.

## Introduction

Research papers play a repository role in recording information on the various elements of science and technology R&D activities. Such elements include information on the source of the research (researchers, research institutes, regions, countries, etc.), the management system of the research (funding information), the communication medium of the research results (source information), science and technology research activities information on social and cognitive connection relationships (co-author information, citation information, etc.), information on various areas and hierarchical structures in which research activities are carried out (science and technology classification information), and information about the contents of knowledge accumulated through research activities (keywords, text), among others. Researchers in the field of scientometrics collate and recombine the above information to measure the impact of the various levels of research performers such as researchers, research institutes, and countries or to grasp the qualitative and quantitative changes of activity and the trends of changes in specific research areas. To those ends, they create geographic knowledge maps based on science and technology activities to identify specific areas of science and technology research and understand the flow of knowledge among them. The method of constructing the knowledge map, including the terrain of research activity, is as follows. 1. Define nodes on the knowledge map, such as journals, researchers, research institutes, countries, keywords and so on, as analysis units. 2. Extract the relationships (links) between nodes from science and technology activities. 3. Cluster the nodes into similar areas using that link information. 4. Identify areas based on the grouped nodes and isolate the directions of science and technology development through time-series analysis. Monitoring of changes in technology clusters over time enables investigation of the following: quantitative changes such as enlargement or reduction of technology cluster size; qualitative changes in research contents such as separation or merging of technology clusters; structural changes such as to connections between internal nodes within clusters or between different clusters. Given these investigative capabilities and the benefits thereof, many studies on monitoring of research-field dynamics have been conducted with the aim of extracting the various indicators related to the dynamics of research areas and predicting, thereby, changes in future growth potentials and their impacts [1–6]. That is, they have mostly extracted various analytical indicators and focused on exploring the relationship between indicator fluctuations and technological growth. However, the extant research in this vein has limitations. First of all, it has been conducted to quantify the development of research fields by focusing on research in a specific field [7–9]. Studies to quantify the development of a research area by focusing on a specific area of research are meaningful in themselves, but there are clearly limitations in that they cannot capture changes within global research areas. Mapping the structure of the entire study area using relationships such as citation relationships between areas allows tracking of the development of detailed areas on this configured map. Secondly, due to the limitations of data processing capacity, clustering and identifying of area codes have been performed based mainly on only core papers such as those that have been the most frequently cited [10–12]. However, an analysis based on a core paper has the disadvantage that it cannot accurately describe its area. Comprehensive analysis of all scientific and technical areas based on the entire literature, by contrast, enables detailed analysis and relative comparisons of specific areas with all areas [13–17]. In this study, we investigated quantitative changes in global technology clusters over time and devised a prediction model for detection of newly emerging or rapidly growing technology clusters. All research areas were considered, utilizing micro-field information published by the Centre for Science and Technology Studies (CWTS) [18] of Leiden University in the Netherlands to guarantee the reliability and reproducibility of the results. The entire literature for analysis was collected in conjunction with Web of Science (WoS) [19]. Recently, the need for meta-knowledge has increased as the amount of accumulated knowledge related to science and research has accumulated and the factors needing to be considered have diversified. Meta-knowledge encompasses various additional information, such as statistical information and information obtained through natural language processing, that facilitates prompt and efficient knowledge acquisition and understanding [20]. Therefore, this study also examined the applicability of various forms of meta-knowledge suitable for detection of rapidly growing technology clusters. The remainder of this paper is organized as follows. Our proposed future-growth-potential prediction model is introduced in Section 2. The promising technology-selection process based on the proposed model, and specific technologies thus selected, are presented in Section 3. Finally, conclusions and recommendations for future study are made in Section 4.

## Future-growth-potential prediction model

### Process of future-growth-potential prediction model

**Fig 1:**
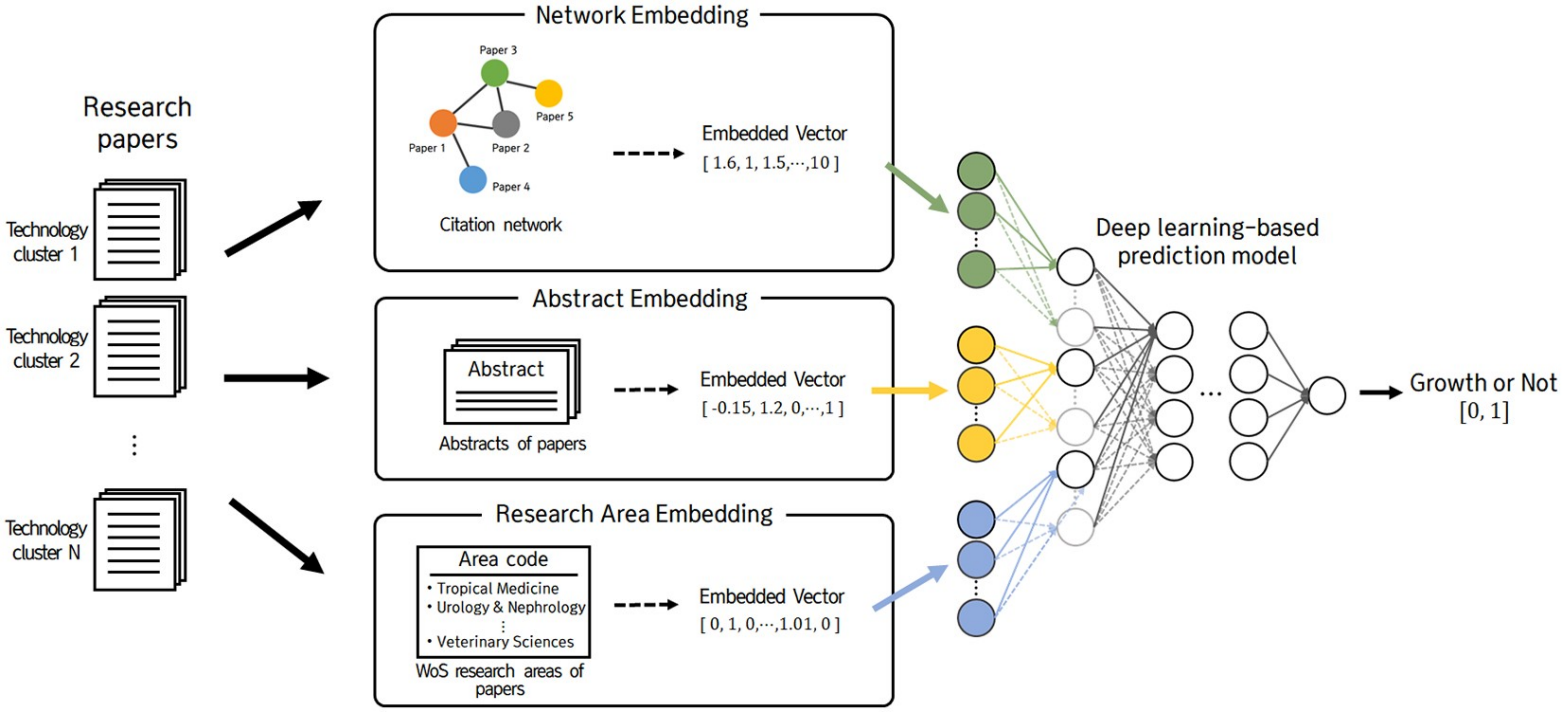
Overview of deep learning model for prediction of future growth potential.

The proposed prediction model consists of three steps: data collection, data embedding, and deep learning-based prediction model training and prediction. Deep neural networks (DNNs) are typically used to model complex nonlinearity of high-dimensional data in regression or classification problems. The proposed model uses deep learning algorithms to convert text data containing the core content of the paper into numerical data in the data embedding step and predicts the future growth potential of the technology in the prediction step. A recent study used deep learning to predict new technologies [21]. Specifically, it used a deep learning algorithm to predict emerging technologies in Gartner’s hype curve in 2017 based on patent data from 2000 to 2016 and overcome the limitations of small samples. In the present study, by contrast, we used deep learning to embed text information (i.e., citations, abstracts, area codes) as meta-knowledge and predict the growth potential of technologies. The process of the future-growth-potential prediction model is shown in Fig 1.

### Data collection

This research is processed by matching the Web of Science database [19] with CWTS micro-level field information [18]. CWTS provides the “Leiden Ranking” that quantitatively analyzes the scientific performances of major universities around the world and the publications’ assignment information in the micro-level field based on bibliographic data from the WoS database. WoS is one of the representative citation databases covering a wide range of international scientific literature generated by Clarivate [19]. For this study, 16,298,856 research papers published from 2006 to 2017 were collected after preprocessing.

**Fig 2:**
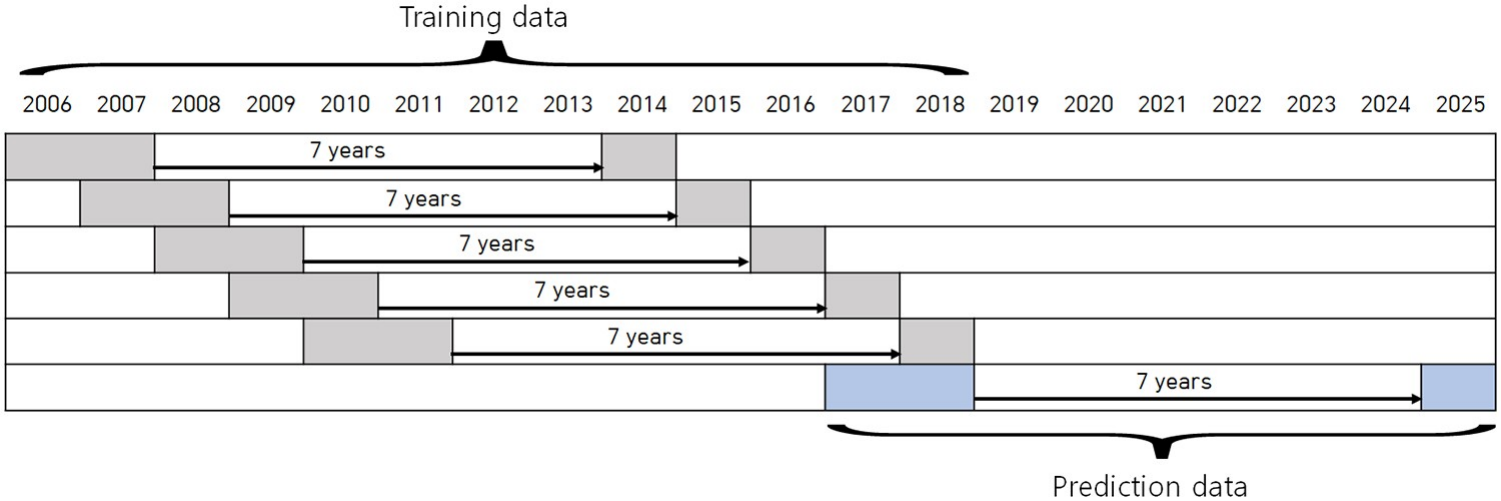
Construction of training and prediction datasets.

Then, reference information, abstracts, and WoS research-area information were extracted from each research paper. The purpose of the deep learning model for prediction of future growth potential is to predict whether a technology cluster will grow after 7 years (2024) based on two years’ worth (2016-2017) of various meta-knowledge (again: citations, abstracts, area codes) extracted from research papers. Among the meta-knowledge, the citations allow for understanding of the cohesiveness and network-structural characteristics among papers belonging to a specific research area; the abstracts are brief summaries of the research papers and cover the essential contents, including the research findings, the key conclusions of the research, and the methods used; the area codes account for the degrees of convergence and diffusion of research categories. To those ends, we constructed four training datasets from the four pairs of data shown in Fig 2 (2006-2007 vs. 2014, 2007-2008 vs. 2015, 2008-2009 vs. 2016, 2009-2010 vs. 2017).

### Data embedding

The embedding vectors to be used as input variables of the deep learning model were generated as follows. For the 4,535 technology clusters’ 4 datasets, three embedding vectors were obtained by performing citation network embedding, abstract embedding, and research-area embedding. The embedded vectors were then used as the input data for the prediction model.

#### 1) Embedding vectors for citation network

**Fig 3:**
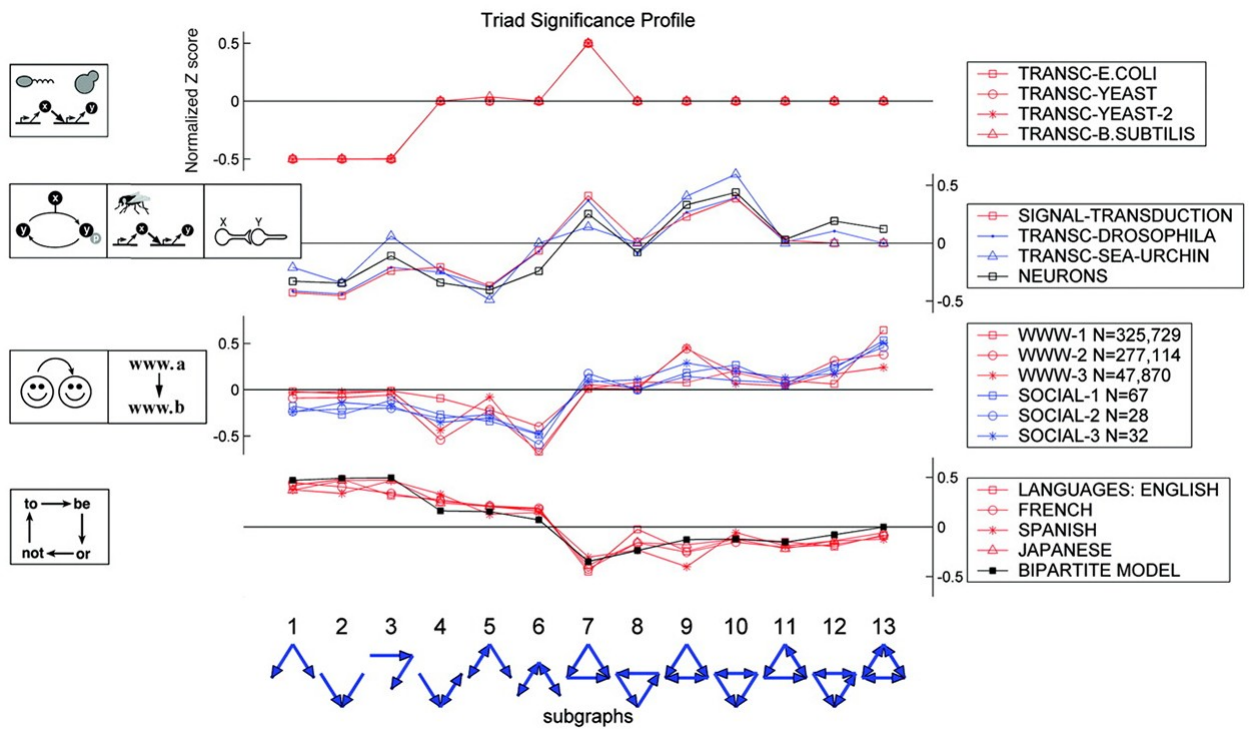
Motif distribution examples detected in various fields of network [23].

**Fig 4:**
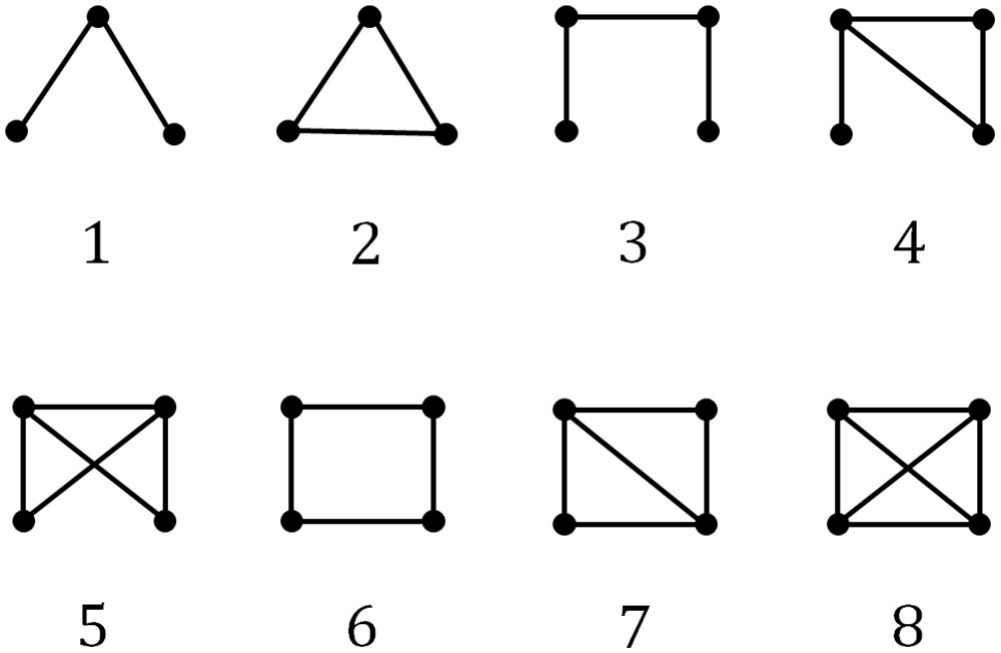
Motifs used to embed citation network.

For the network embedding of the technology clusters, the citation network was constructed with research papers belonging to each technology cluster, and then the embedding vector was obtained based on the motif representing the network as a distribution of subnetworks. To construct the citation network, we calculated the cosine similarities among the research papers within each cluster using a bibliographic coupling method and extracted a non-directional binary citation network with 1 if the similarity value was equal to or greater than 0.3, and 0 otherwise. Then, by using the distribution information of the motif subnetworks in the citation network of each cluster, it was possible to grasp the structural characteristics of the networks and measure the structural similarities among those networks. A motif is a subnetwork that is found more frequently in a network than are random networks of the same size [22]. The motif is one of the important attributes that reflect the functional characteristics of a network, and is a useful concept for exploring the principles of the structure of complex networks. If the frequency of expression of subnetworks in a complex network is above average, it is interpreted as having special functions and meanings. As shown in Fig 3, networks of the same type have similar characteristic motif values that can be used to understand the characteristics of any of those networks. In general, the measured frequencies of real motifs are expressed by normalization to the frequency of motifs in the random network in order to find their relative importance. The network used in this study is a non-directional binary network; therefore, a distribution of eight motifs consisting of three and four nodes was used as shown in Fig 4. In this study, we assumed that the growing technology clusters will have similar motif distributions in the citation-similarity networks.

#### 2) Embedding vectors for abstracts

The abstract of each technology cluster was embedded as a vector using bidirectional encoder representations from transformers (BERT) developed by Google. BERT is a language representation model that shows very good performance in various natural language processing problems. Since BERT already has a pre-trained model obtained by training based on a large amount of text data, it was possible to embed abstracts of research papers within technology clusters using the pre-trained model [24]. The BERT model is composed of several encoders of the transformer model, which is trained to output embedding vectors of specific words according to the context [25]. The transformer model is a machine translation model that models sequence data using the attention technique, which outperforms the recurrent neural network (RNN) model. Whereas the RNN model processes input data sequentially, the transformer model has a structure in which multiple encoder layers and decoder layers are stacked, and it is designed to process a given sequence at once to shorten learning time. On the other hand, since the BERT model is pre-trained by predicting deleted words and predicting the relationship between sentences, it can perform embedding of words by considering the context before and after, and it can identify the relationship between words as well as sentences. Using BERT’s pre-learning model, high-performance embedding vectors for input sentences can be obtained. Therefore, the abstracts of the papers belonging to each technology cluster were embedded with the pre-trained model, and then the mean vector of all papers belonging to each cluster was used as the embedding vector of the technology cluster. As a result, a 768-dimensional abstract embedding vector was extracted for each technology cluster by year.

#### 3) Embedding vectors for area codes

Each paper in the WoS database was classified into one of a total of 256 research categories [19]. Therefore, the category frequency distribution of research papers per technology cluster was calculated and normalized so as to embed the area codes for each technology cluster. As a result, a 256-dimensional area-code embedding vector was extracted for each technology cluster by year.

### Deep learning model

#### 1) Deep learning structure and training

**Fig 5:**
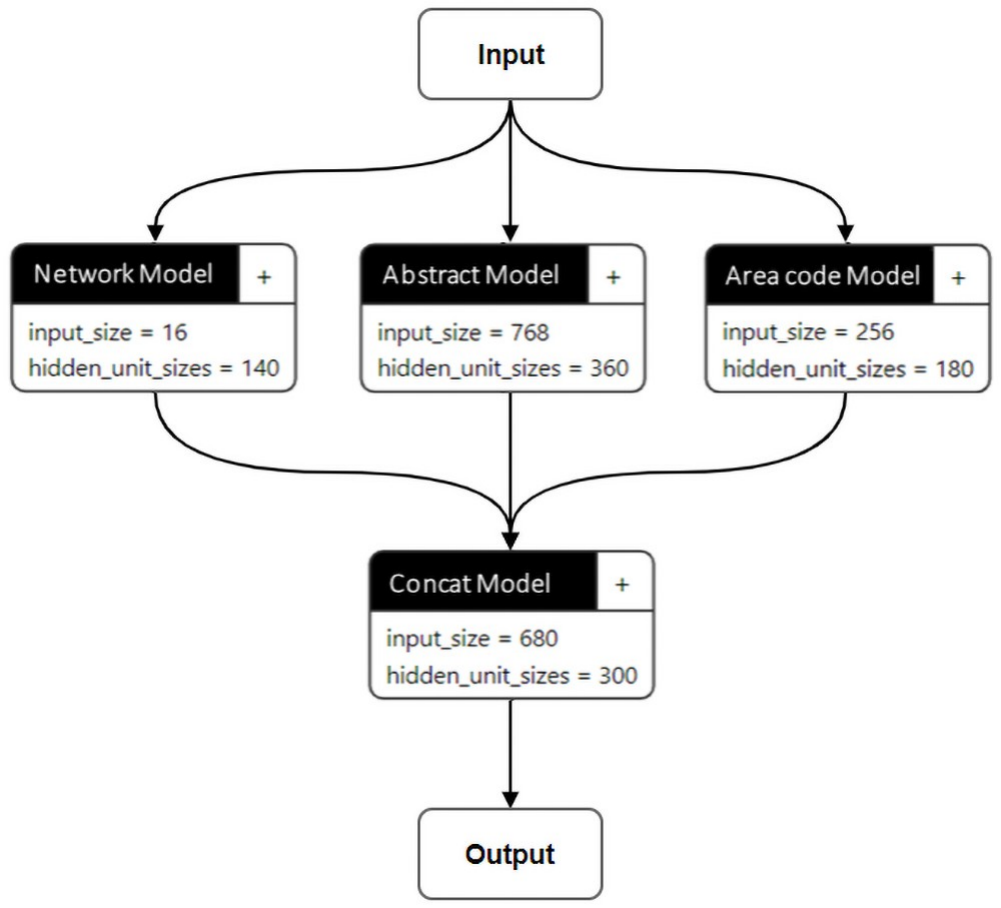
Optimal deep learning structure for prediction of future growth potential of technology clusters.

Deep learning has a feature that models the complex nonlinear relationship between input and output data [26]. We used the deep learning model to predict future growth potential because it has shown good performance in many fields recently [27, 28], and at the same time, it yields the probability that each observation will be classified into a class. In this study, the probability was redefined as the future growth potential of each technology cluster, that is the probability that each cluster will be classified into the growth class, as predicted by the deep learning model. We built a model for prediction of the future growth potential of each technology cluster based on deep learning incorporating, as input variables, the embedded citation network, abstract and area-code vectors of the technology cluster. The derived 16-dimensional citation network embedding vector, the 768-dimensional abstract embedding vector, and the 256-dimensional research-area embedding vector were combined to form one input vector for each technology cluster of each year. However, the two-year citation network embedding vectors were used as input vectors to account for the trends of the citation network. Consequently, each input vector consisted of a 16-dimensional network embedding vector, a 768-dimensional abstract embedding vector, and a 256-dimensional research-area embedding vector for each technology area of each year. The target value of the deep learning model was not determined based on the absolute criteria for the individual technology cluster but rather on the relative position of the individual technology cluster in the distribution of growth rates calculated for all technology clusters. For the target value, we estimated the slope of the trend line with respect to the logarithm of the number of papers belonging to each technology cluster using least-squares regression analysis, and then defined the classes (i.e., growth and non-growth) of the technology clusters based on the top 30% slope (0.06545). In order to capture the exponential growth pattern of the number of papers belonging to each technology cluster, the technology growth was calculated using the logarithm of the number of papers, and the slope of the trend line was used to consider the change in the number of papers over 7 years. The optimal deep learning structure derived by finding the optimal parameters through repeated experiments is shown in Fig 5. The hyper-parameters of the deep learning models included the number of layers, the number of nodes per layer, the learning rate, and the epoch. As seen in Fig 5, the deep learning structure has a structure that combines three models based on the embedded values of the citation network, abstracts, and area codes. Each model for the citation network, the abstracts, and the area codes have one hidden layer. The optimal number of hidden nodes for the models of the citation network, the abstracts, and the area codes was 140, 360, and 180, respectively. The outputs for the models of the citation network (size 140), the abstracts (size 360), and the area codes (size 180) are concatenated and used as input vector (size 680) for the combined model. The combined model consists of one hidden layer with 300 optimal hidden nodes and the last softmax layer that outputs the probability of each technology cluster being classified into the growth class. The deep learning model is optimized in an end-to-end manner. To train the prediction model, RAdam (Rectified Adam) [29] for the optimizer, Binary Cross Entropy (BCE) for the loss function, ReLU for the active function, and the learning rate = 0.00007 were used. In addition, we used a dropout method with a drop ratio of 5% to avoid the overfitting problem and BCE with a weight of 2.4 times for class 1 (growth) to solve the imbalance problem of the training data.

#### 2) Evaluation results of deep learning model

To evaluate the generalization performance of the deep learning model, we compared the performance of the proposed model to those of methods in wide use, including logistic regression [30], SVM [31], random forest [32], deep learning which uses the combined three embedding vectors as input data. For comparisons, we used 5-fold cross-validation, dividing the data into training data and validation data five times. The procedure of 5-fold cross-validation is as follows. First, the entire data are divided into five subsets of similar size, and the first experiment learns using the first subset as the validation data and the remaining subsets as the training data. In the second experiment, the second subset is used as validation data and the remaining subsets are used as training data. In the same way, the model is evaluated based on the average performance value of the validation data derived from a total of five experiments. The performance of the trained deep learning model was evaluated according to the accuracy of the classes (i.e., growth vs. non-growth) of technology clusters and the F1 measure based on the results predicted by the deep learning model. The accuracy and F1 measure are defined as follows.

$$\begin{array}{c} Accuracy=\frac{number\;of\;correctly\;predicted\;technology\;clusters}{total\;number\;of\;predicted\;technology\;clusters} \end{array}$$

$$\begin{array}{c} F1=\frac{2\;\cdot \;Precision\;\cdot \;Recall}{Precision\;+\;Recall} \end{array}$$ (1)

F1 is the harmonic mean of precision and recall. The precision and recall in Eq (1) are defined as follows.

$$\begin{array}{c} Precision=\frac{number\;of\;technology\;clusters\;actually\;grown(not\;grown)}{number\;of\;technology\;clusters\;predicted\;to\;be\;grown(not\;grown)} \end{array}$$

$$\begin{array}{c} Recall=\frac{number\;of\;technology\;clusters\;predicted\;to\;be\;grown(not\;grown)}{number\;of\;technology\;clusters\;actually\;grown(not\;grown)} \end{array}$$

The accuracy and F1 for growth and non-growth are summarized in Table 1. F1 for growth is an F1 measure for the technology cluster with growth class, and F1 for non-growth means the F1 measure for the technology cluster with non-growth class. From Table 1, we observe that the proposed deep learning model performs better than the other conventional methods. Note that the proposed model yields better results than the deep learning model using the combined three embedding vectors as input data. It shows that properly combining vectors into the latent feature space is more important than combining them in the input space. For the proposed deep learning, the accuracy, the F1 measure for growth, and the F1 measure for non-growth was 0.8672, 0.7428, and 0.9105, respectively.

**Table 1:** Performance of prediction models.

Models	Accuracy	F1 for Growth	F1 for Non-Growth
Logistic Regression	0.6592	0.5091	0.7389
SVM	0.6892	0.4272	0.7867
Random Forest	0.7472	0.4119	0.8390
Deep Learning	0.8055	0.5841	0.8730
Proposed Deep Learning	**0.8672**	**0.7428**	**0.9105**

According to Fye et al. [33], who measured the success rate for each prediction method applied to 295 verified cases of technology prediction, the method with the highest success rate was a quantitative trend analysis method, with the success rate of 64.3% and the realization rate of 67.9%. Meanwhile, the technique based on experts had a success rate of 38.3% and a realization rate of 75.7%. The average success rate of each prediction technique was 36.9%, and the average realization rate was 66.1%. The techniques that combined the views of several experts showed the highest probability that the predicted technology would be realized, but the prediction accuracy of the realization time was low. On the other hand, in the case of the quantitative measurement technique, the prediction of the realization was rather low, but the prediction of the realization time was the highest. The realization rate of predictions discussed in Fye et al. [33] and the growth potential of the present study are similar. Therefore, the results of the present study, shown in Table 1, can be said to be competitive when compared to other prediction methods including the expert-based technique.

## Promising technology selection

### Promising technology selection process

**Fig 6:**
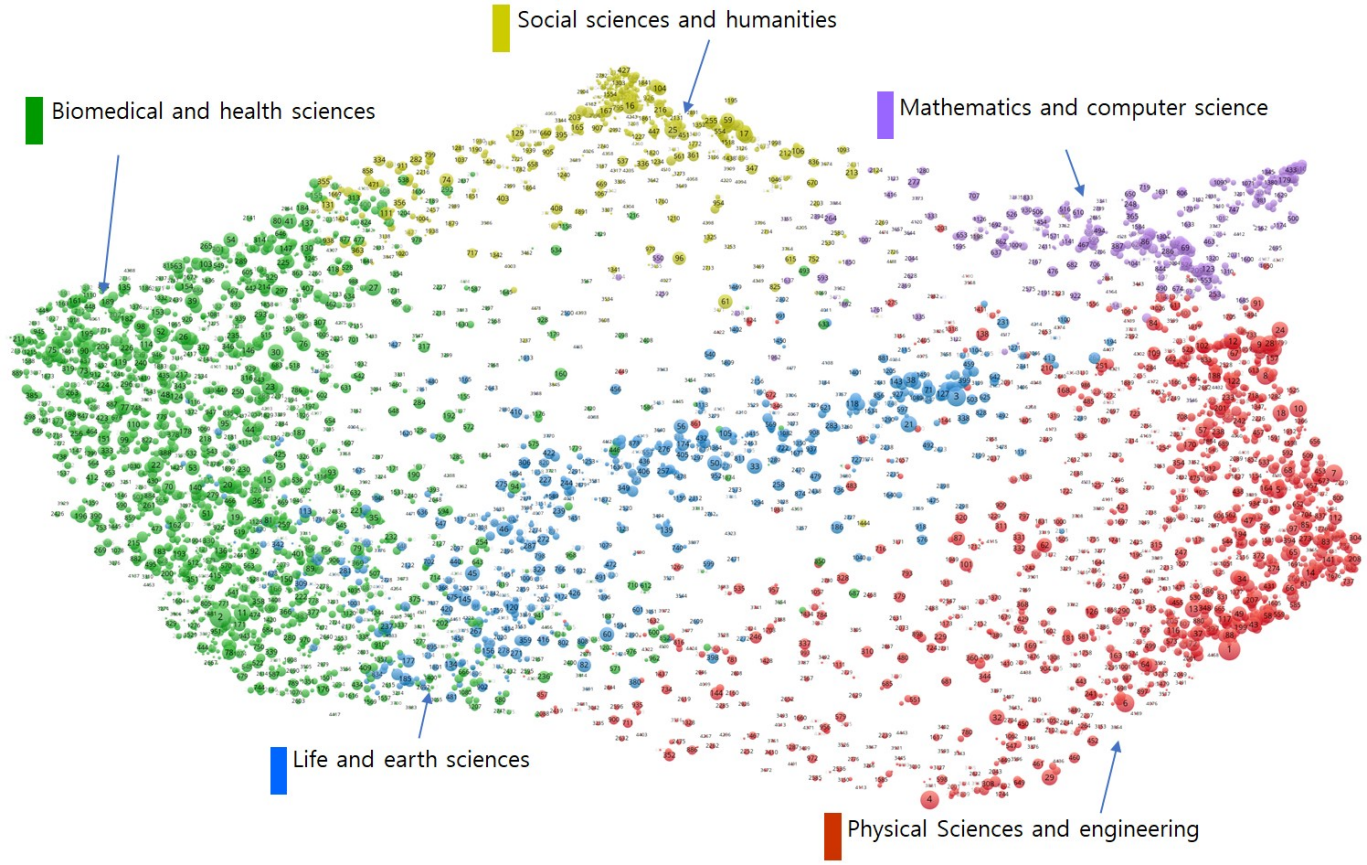
Similarity-based mapping of all technology clusters.

**Fig 7:**
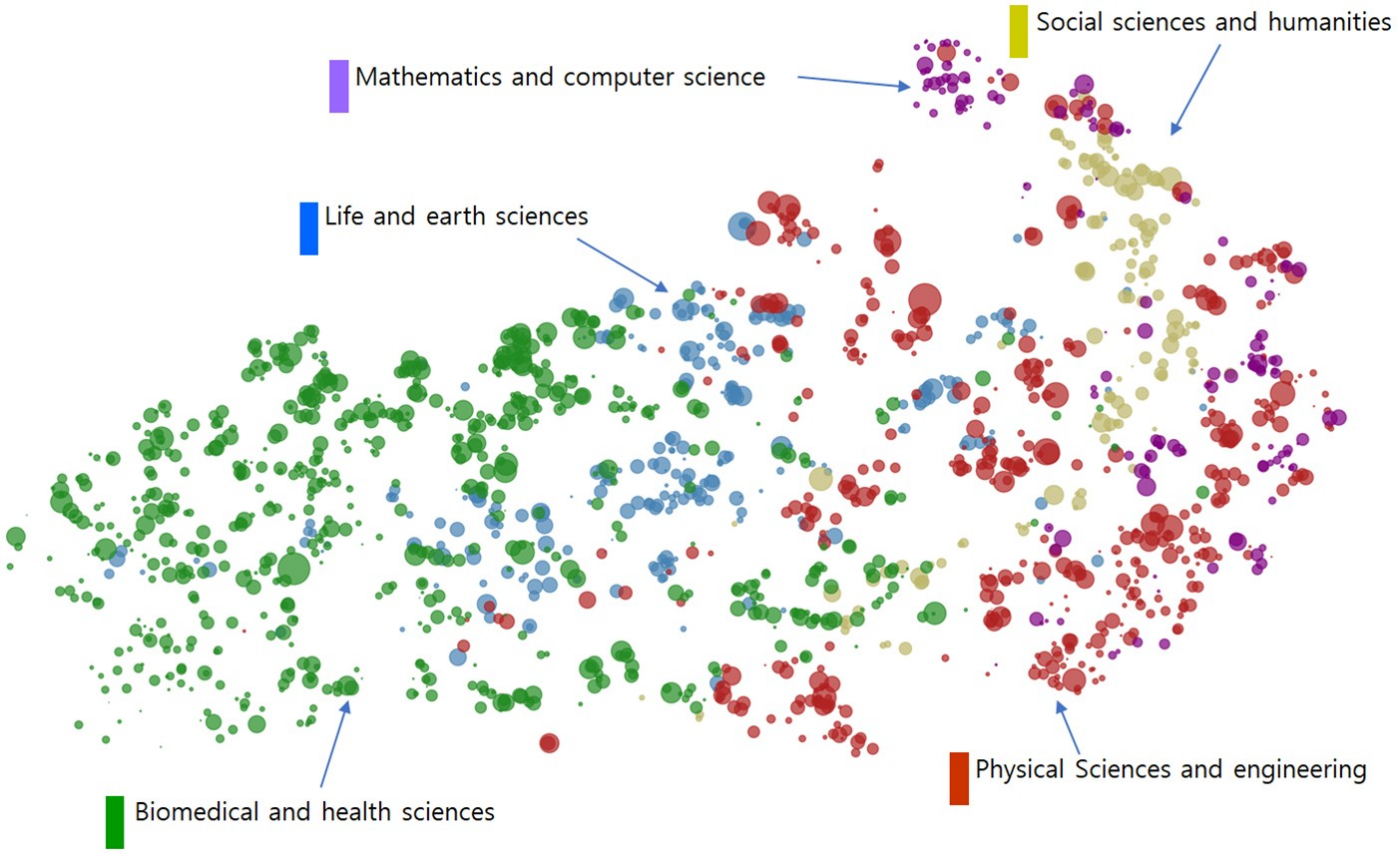
Abstract-based mapping of all technology clusters.

First, in order to grasp the relationship among all of the technology clusters, the mappings of technology clusters were performed based on their similarities and abstracts. Those similarities were calculated by the following procedure. For each article in each cluster technology, the classification codes (256 WoS categories) for references in each article were investigated, and then a 256-sized frequency vector for each article was obtained. The vector of each technology cluster was then derived by summing the vectors for the individual articles belonging to each technology cluster. Then, the cosine similarities of the vectors among the entire 4,535 clusters were calculated. The mapping results based on the 4,535 x 4,535 similarity matrix in Fig 6 were obtained using VOSviewer software [34]. As seen in the figure, all of the technology clusters were within the five main fields, which were mathematics and computer science, physical sciences and engineering, life and earth sciences, biomedical and health sciences, and social sciences and the humanities. In the map, each technology cluster in CWTS is colored differently according to the field. In addition, the abstract-based mapping of all technology clusters was performed based on the 768-dimensional abstract embedding vectors using t-distributed Stochastic Neighbor Embedding (t-SNE) algorithm [35]. The t-SNE algorithm visualizes high-dimensional data in two dimensions, and the mapped data points (i.e., technology clusters) can be clustered based on their similarities. Therefore, t-SNE algorithm can be a useful tool for automatic visualization and clustering of large data sets [36]. Like the similarity-based map, each technology cluster in CWTS has a different color depending on the field. As can be seen in Fig 7, not all technology clusters are clearly classified, but most technology clusters have been roughly classified into five major fields, similar to the similarity-based map. These results indicate that while abstract embedding can be an important meta-knowledge that well reflects the semantic of technology clusters, other meta-knowledges are additionally needed to predict future growth potential.

**Fig 8:**
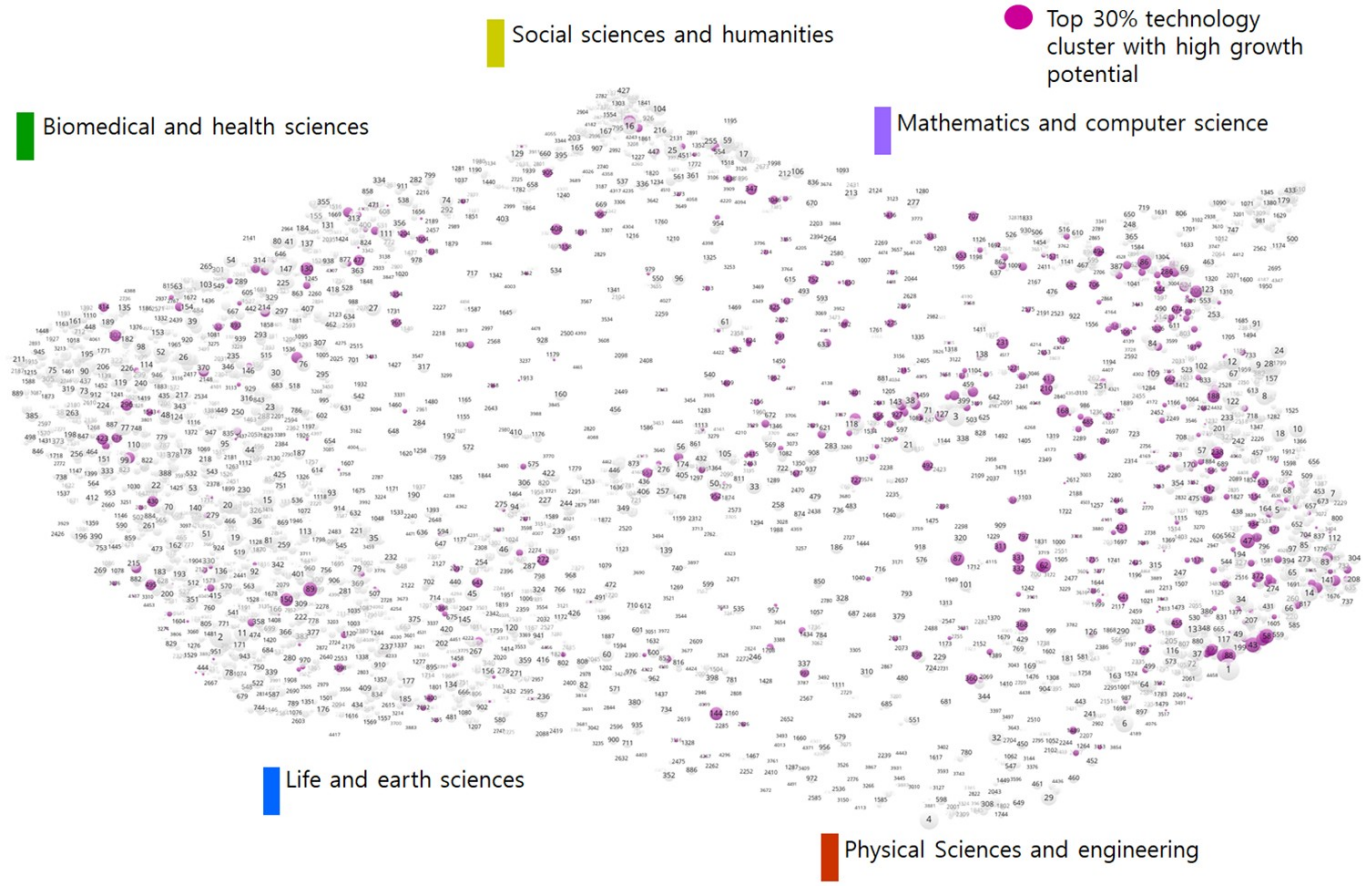
Technology clusters with high growth potential (purple nodes).

**Fig 9:**
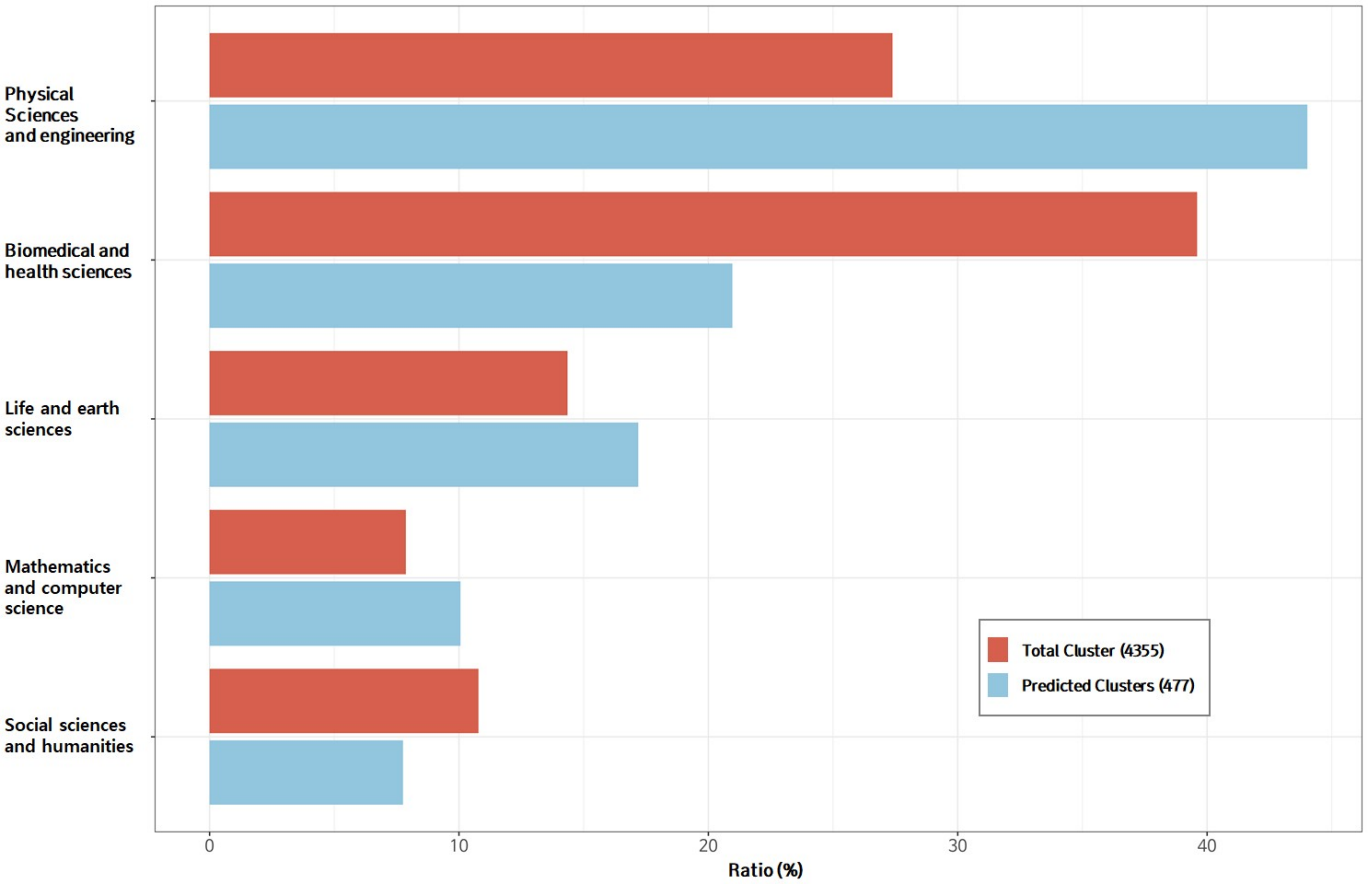
Five fields’ technology clusters: Ratio of number of field’s clusters to total clusters (red color); Ratio of number of field’s clusters to clusters predicted to have high growth potential (blue color).

Then, by applying the deep learning-based future-growth-potential prediction model to the total of 4,535 technology clusters, we selected the promising technology candidates with high seven-years-later growth potential. The 477 technology clusters thus selected by the deep learning model are shown in Fig 8’s mapping results. The five main fields’ technology cluster ratios in both the total 4,535 clusters and the 477 clusters predicted to grow in seven years are plotted in Fig 9. As can be seen, the biomedical and health sciences field’s ratio in the total 4,535 clusters was close to 40%, but fell to about 20% relative to the 477 clusters predicted to grow. The physical sciences and engineering field had the highest ratio of clusters relative to the 477 clusters predicted to grow, about 48%. Likewise, the life and earth sciences and mathematics and computer science fields’ technology clusters’ ratios also were higher relative to the 477 predicted-to-grow clusters than to the total 4,535 clusters. Meanwhile, the opposite trend was seen for the biomedical and health sciences and social sciences and humanities fields.

**Fig 10:**
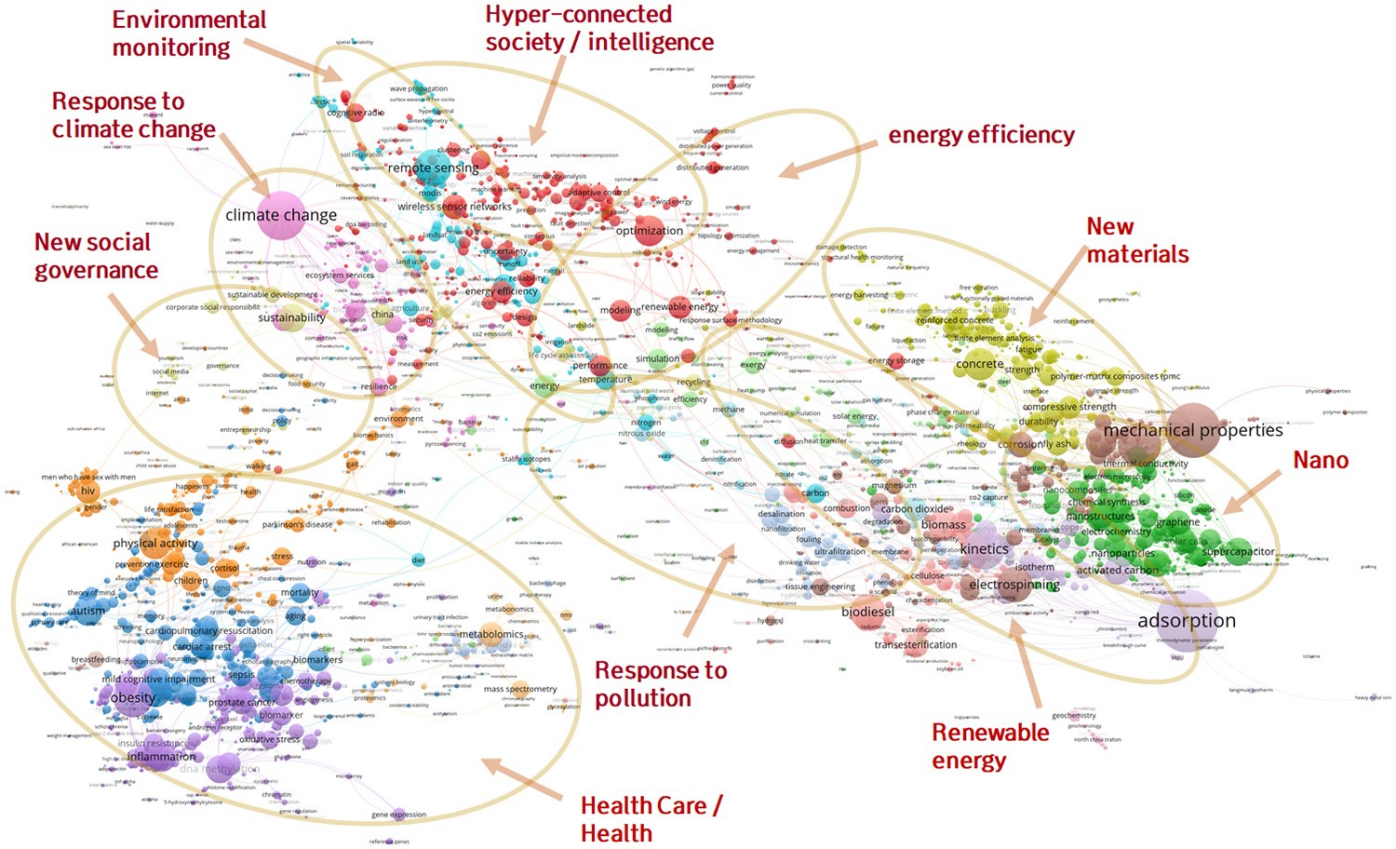
Keyword mapping of technology clusters with high growth potential.

In order to identify the overall trends of the 477 technology clusters with high potential for future growth, mapping analysis based on keyword co-occurrence frequency was performed, as shown in Fig 10. There were 1,738,632 references in the 477 technical clusters with high growth potential. There were 1,437,112 author keywords in the references, and 4,758 keywords that appeared in more than 20 references simultaneously were extracted from 7,716 keywords that appeared more than 100 times. Among them, mapping analysis was performed on the 4,598 keywords that made up the giant component. The colors of the nodes in Fig 10 were assigned in the clustering analysis by the co-occurrence link in VOSviewer. In the figure, we can see, by referencing the links between keywords, the macro trends driving the growth of technologies. At the bottom left, there is a huge trend named ‘health care / health’. On the right is a huge ‘materials’ trend including the ‘nano’, ‘new materials’ and ‘renewable energy’ trends. There are also, penetrating from the top left and continuing to the bottom right, huge ‘environment’ trends including ‘response to pollution’, ‘environmental monitoring’ and ‘response to climate change’. On the other hand, operations-related trends such as ‘hyper-connected society / intelligence’, ‘new social governance’ and ‘energy efficiency’ can be identified in conjunction with other related giant trends.

**Fig 11:**
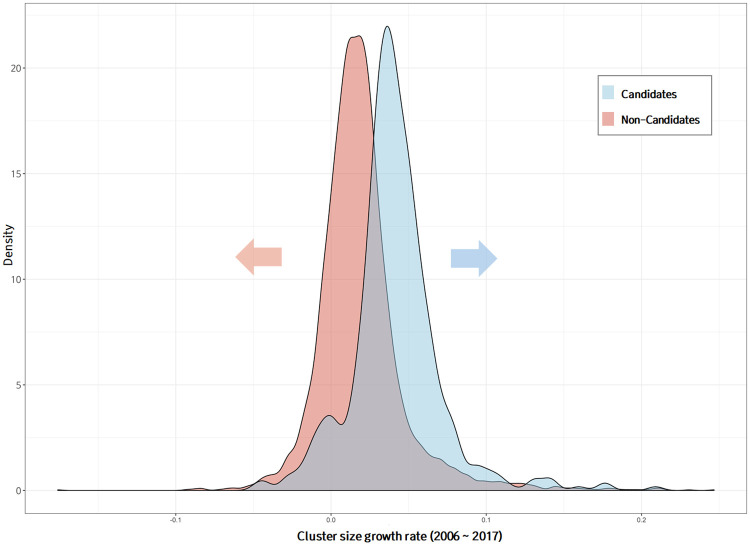
Comparison of growth rate distribution of technology clusters with and without high growth potential over all periods.

**Fig 12:**
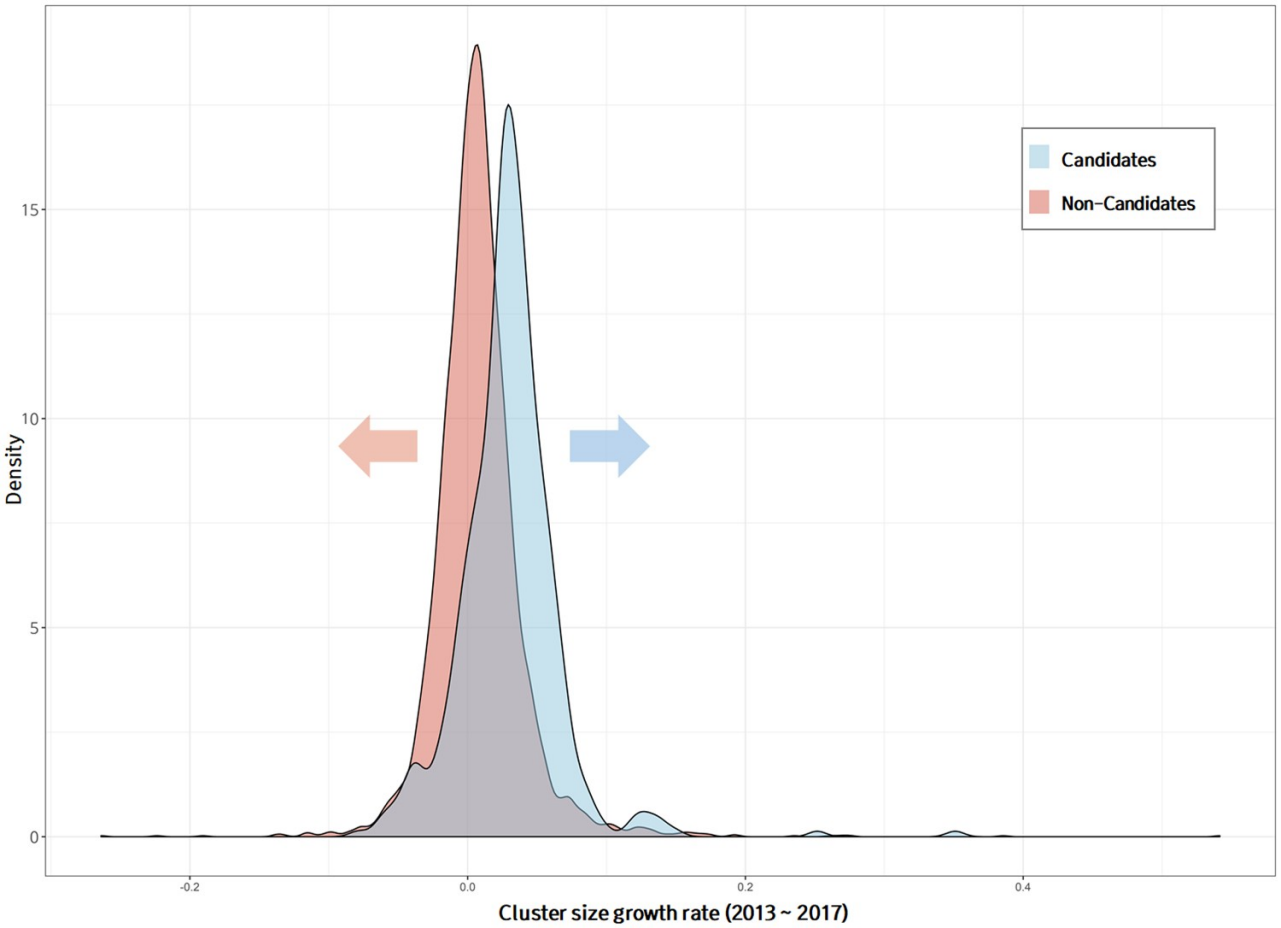
Comparison of growth rate distribution of technology clusters with and without high growth potential over last 5 years.

To investigate the difference between technology clusters with high growth potential and those without, the entire group of technology clusters was divided into the technology clusters with high growth potential [477 candidate groups] and the remaining clusters [non-candidate groups]. Figs 11 and 12 show the distribution of the cluster size growth rate during the entire analysis period [2006-2017] and the last five years [2013-2017], respectively. Candidate groups predicted to have a high growth potential had higher average growth slopes than did the non-candidate groups over the entire period and the last five years. To evaluate the difference between the means of the two populations, the hypothesis t-tests were conducted. A p-value is used in hypothesis t-test to decide whether to reject the null hypothesis (i.e., *H*_0_: *μ*_1_ − *μ*_2_ = 0). The p-value is a probability to measure the evidence against *H*_0_ provided by the sample [37]. Smaller p-values indicate more evidence against *H*_0_. As shown in Figs 11 and 12, the average growth slopes between the candidate and non-candidate groups were different for the entire period and the past 5 years, and the p-value for each t-test was less than 0.001. However, the distributions of the candidate and non-candidate groups overlapped considerably. The variables used as input in the deep learning model included network embedding, keyword embedding, and research-area embedding vectors, which did not contain any information related to the past growth trends of the clusters. Therefore, the cluster growth trends could be used as an additional factor for screening technology clusters with high growth potential. Our forecasting model focused on the growth of technology, and so a variety of perspectives and criteria related to promising concepts were not taken into account. In particular, the aspect of future market value was not considered at all. Additional, market- or industry-related criteria may be applied to the selection of promising technologies, but were beyond the scope of this study.

Among the first candidates selected by the deep learning-based prediction model, the final candidates were selected by applying additional criteria related to the growth trends of the technology clusters. First, the probability of growth in the future, which was the criteria for the first candidates, was somewhat relaxed from 99.5% to 99.0%, increasing the number of candidates to 604. Among the selected candidates, 43 technical clusters, whose main field was social sciences and humanities, were excluded. The additional criteria reflecting the growth trends of the technology clusters were set as follows.

1. Technology cluster size: The number of studies in the selected technology cluster from 2000 to 2018 should exceed 3,900, which is the median of the total technology cluster size.
2. Rise of technology cluster: The growth rate of the last 5 years (2013-2017) of the selected technology cluster should be higher than that of the last 12 years (2006-2017).
3. Latestness of technology cluster: The average age of studies in the selected technology cluster should be less than the 25th percentile (i.e., 2011.3) of the average age of all of the technology clusters’ studies.

A total of 24 candidates were selected by applying those additional criteria, and the final 10 technologies were evaluated and selected by conducting the evaluation based on the criteria of technical ripple effects, compatibility with social issues, and government policy compliance.

### Ten promising future technologies

The 10 promising future technologies selected are shown in Table 2, where the number of studies is the number of papers published between 2000 and 2018, the growth rate is the log slope of the number of papers from 2013 to 2017, the age is the average publication age of the papers belonging to the cluster, and the CWTS ID is the unique number of cluster provided by CWTS. The most prominent trends related to the 10 technologies shown in Fig 10 were energy efficiency, hyper-connected society/intelligence, and environment. The fact that no technology corresponding to the health care/health trend appeared among the 10 technologies was due to the second screening criteria. Among the first candidates, there were 100 technology clusters belonging to the biomedical and health sciences field; however, the technology clusters in the field of biomedical and health sciences had low average slopes in the last five years and high publication ages, and so most of them were removed in the course of the second screening process. Note that our purpose in this study was not to pick out the 10 final, objectively promising technology clusters. The criteria applied to the selection of the technology clusters are not absolute; thus, the 10 selected technology clusters could be changed if different criteria chosen according to the analyst’s personal perspective or specific purpose were applied.

**Table 2:** Performance of deep learning-based prediction model.

Name of technology cluster	Number of studies	Growth rate	Age	CWTS ID
Renewable energy storage and conversion technology utilizing hydrogen energy	5,288	0.0996	2012.0	1709
Next-generation eco-friendly heating and cooling system core material technology	5,908	0.1324	2011.8	1495
Carbon dioxide capture and storage technology	9,938	0.2164	2013.2	1489 (2173)
Advanced autonomous vehicle technology	3,949	0.0967	2011.7	2197
AI-based machine vision technology	4,296	0.1227	2012.1	2040
Ultra high performance concrete technology	5,771	0.0912	2011.4	1544
Biodiversity research	10,487	0.1012	2011.7	557
High-voltage, direct current (HVDC) technology	14,385	0.1460	2012.5	209
Humanoid robot technology	4,807	0.0879	2011.6	1862
Hyperspectral imaging technology	13,964	0.1243	2012.4	231

## Conclusion

This study developed a deep learning model for prediction of the future growth potential of technologies and used it to select 10 promising technologies. The key question addressed in this paper was whether it is possible to predict the future growth potential of technologies based on data regarding the relevant respective research activities. To answer this question, the embedding vectors for the citation network structure within the technology cluster, the subject structure obtained from paper abstracts, and the area codes were used as input variables in the prediction model. Utilizing this meta-knowledge, the deep learning-based prediction model showed more accurate performance and correspondingly high potential. There is, in fact, a need for a methodology and framework that can complement data-driven and expert-driven predictions. If data-driven predictions are highly accurate, data analysis results can be provided as objective evidence to reduce the subjectivity of the intervention of experts and improve overall forecasting accuracy thereby. Conversely, experts’ insights on the directions of future technologies should be incorporated into data-driven forecasting methods to improve them as well. In other words, it is necessary to further study and take advantage of the virtuous cycle by which the results of data-based prediction methods are subjected to expert interpretation, the results of which are again utilized for data-based prediction methods. Going forward, it will also be necessary to improve the understanding of deep learning-based prediction by applying explainable artificial intelligence (AI) algorithms, which in turn will deepen the understanding of the structure and characteristics of science and technology research activities.
